# Efficient Degradation of Carbamazepine in Continuous and Batch Modes by Laccase-Photo-Fenton-Intensified Hybrid Treatment

**DOI:** 10.1021/acsestwater.5c00676

**Published:** 2025-11-24

**Authors:** Natalia Klanovicz, Pratishtha Khurana, Bruno Ramos, Helen Treichel, Satinder Kaur Brar, Antonio Carlos Silva Costa Teixeira

**Affiliations:** a Research Group in Advanced Oxidation Processes (AdOx), Department of Chemical Engineering, Escola Politécnica, 67420 Universidade de São Paulo, São Paulo 05508-080, Brazil; b Department of Civil Engineering, Lassonde School of Engineering, 7991 York University, Toronto, ON M3N 3A7, Canada; c Department of Chemical Engineering, Centro Universitário FEI, São Bernardo do Campo 09850-901, Brazil; d Laboratory of Microbiology and Bioprocesses, Universidade Federal da Fronteira Sul, Erechim 99700-000, Brazil

**Keywords:** organic contaminant, treatment intensification, microdevices, design of experiments, degradation pathway, ecotoxicity prediction

## Abstract

Despite advances in the removal of pharmaceutical residues from aqueous effluents, carbamazepine (CBZ) remains challenging due to its persistence. The low removal efficiency of conventional wastewater treatments reinforces the need to develop innovative approaches, such as hybrid systems. This study combined photo-Fenton reactions with the enzyme laccase (Lac) to effectively remove CBZ from aqueous solutions in batch and continuous-flow regimes. Lac was immobilized on functionalized magnetite nanoparticles (MNPs) to improve stability and operational efficiency. Investigation of the effects of pH, temperature, UVC radiation, and H_2_O_2_ dose on Lac activity revealed promising results. Immobilized Lac retained 77.7% of its initial activity after 60 min of UVC exposure. In contrast, the free enzyme lost its activity within 30 min of exposure. In batch mode, the Lac-MNPs/UVC/H_2_O_2_ system with 2,2’-azino-bis­(3-ethylbenzothiazoline-6-sulfonic acid) diammonium salt (ABTS) as the inducer degraded 91.9% of CBZ in 15 min of reaction at neutral pH. For continuous operation mode, optimization based on a Central Composite Rotatable Design achieved 91.1% CBZ removal at 10 min space-time, 20:1 H_2_O_2_:CBZ molar ratio, and 30 μmol L^–1^ ABTS. The high removal efficiency in both batch and continuous modes indicates the potential application of the developed hybrid laccase-photo-Fenton treatment for effective CBZ degradation.

## Graphical abstract

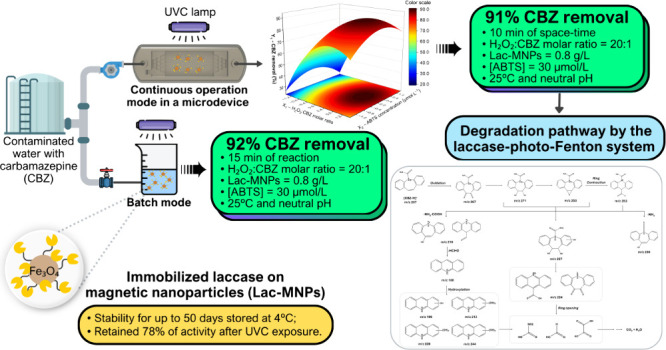

## 1 Introduction

The drugs used for epilepsy treatment include over 25 compounds with varied structures and pharmacological properties.^1–3^ Among them, carbamazepine (CBZ) is one of the most frequently detected anticonvulsant drugs in the environment, being detected in all continents and aqueous matrices.^4^ Studies indicated the occurrence of 442 mg L^–1^ and 0.6 mg L^–1^ CBZ in India and Canada, respectively, from manufacturing wastewater,^5,6^ 288 μg L^–1^ in France from urban wastewater, 170 μg L^–1^ in The Netherlands from surface water,^7^ and 96 ng L^–1^ in the USA from groundwater.^8^

High concentrations of active pharmaceuticals are often released from industrial processes due to washdowns of floors, equipment, and spills.^9^ This release can significantly contribute to their high occurrence in wastewater treatment plants (WWTPs), thereby overloading treatment systems, especially those based on biological processes.^10–12^ Another concern is the detection frequency of this compound in wastewater samples. A survey of samples collected hourly at a municipal WWTP in Portugal showed a 100% detection frequency for CBZ.^13^ In Brazil, CBZ was detected in 100% of the samples collected at five municipal WWTPs before and after each treatment step, which involved different treatment configurations.^14^

Studies that monitored the inlet and outlet CBZ concentrations in conventional treatment plants pointed to alarmingly low removal efficiency, and several cases of negative mass balances. For example, – 92% of removal was observed in a Water Reclamation Facility located at Oak Creek (USA), – 46% and – 64% in WWTPs in Beijing and Xiamen (China), and – 53% in Sewage Treatment Plants located in Southern England. The negative removal of CBZ was found to be of global prevalence and independent of the treatment setup.^14–20^

Employing advanced and innovative degradation processes, such as hybrid treatment systems, is imperative for removing CBZ from contaminated water.^21^ According to Saidulu et al.,^22^ combining treatment processes for removing pharmaceuticals from wastewater presents scale-up potential. Advanced oxidation processes (AOPs) are promising for these hybrid systems, as they comprise a wide range of technologies capable of generating reactive oxygen species with rapid chemical reactions.

The photo-Fenton process is an example of an efficient AOP that can simultaneously degrade pollutants and inactivate pathogens by UVC irradiation.^23–25^ A challenge in this process is the use of soluble iron for Fenton-like reactions, which precipitates at neutral pH. Using iron-containing catalysts, such as magnetite nanoparticles (MNPs), offers a potential solution since these particles are recoverable and biocompatible.^26,27^

The degradation process can be intensified by employing oxidoreductase enzymes, which act as complementary oxidizing agents and are strong candidates to be combined with AOPs.^22,28,29^ Peroxidases (POD) and laccases (Lac) have been extensively studied for their ability to degrade pharmaceutical pollutants,^30–33^ whose principle of action is based on the catalysis of redox reactions responsible for the biotransformation of pollutants. Laccase requires only molecular oxygen to exert its redox action and oxidize persistent contaminants.^30,34,35^ However, the application of free enzymes is limited by their low stability and loss of biocatalyst after use. Therefore, enzyme immobilization offers a solution, allowing the design of reactors for continuous flow operation. Additionally, this downstream process can improve removal efficiency by preventing the rapid saturation of enzymatic and adsorption sites.^36–38^

Therefore, there are significant opportunities for using immobilized laccase with the photo-Fenton process to degrade carbamazepine, as this combination has not yet been evaluated in a single reactor system. This hybrid approach can be implemented as a polishing step in WWTPs, combining the disinfection action of UVC light with the degradation action of reactive species generated from combined enzymatic and photo-Fenton reactions.^39,40^

The aim of this work was to study unexplored aspects of laccase resistance and its application in a hybrid enzymatic-photo-oxidative treatment for water contaminated with carbamazepine. To this end, laccase was first immobilized on MNPs surfaces and characterized, then applied to degrade carbamazepine in a 3D-printed flat-plate reactor operated in continuous flow mode.

## 2 Materials and Methods

### 2.1 Chemicals and Reagents

Carbamazepine (CBZ, C_15_H_12_N_2_O, 236.27 g mol^–1^, CAS 298–46–4), *Trametes versicolor* laccase (Lac, ≥ 0.5 U mg^–1^), acetonitrile, formic acid, (3-aminopropyl)­tiethoxysilane (APTES), and 2,2’-azino-bis­(3-ethylbenzothiazoline-6-sulfonic acid) diammonium salt (ABTS) were acquired from Millipore Sigma (Ontario, Canada). Urea, NaOH, FeSO_4_, Fe_2_Cl_3_, ethanol, glutaraldehyde (GA), and hydrogen peroxide (H_2_O_2_) were purchased from Fischer Scientific (Canada). All solutions were prepared using pure water (18.2 MΩ cm) from a Milli-Q Direct-Q system (Merck Millipore).

### 2.2 Enzyme Immobilization on Magnetite Surfaces

The synthesis of MNPs followed the procedure described elsewhere.^41^ In brief, 100 mL of Fe_2_Cl_3_/FeSO_4_ solution (2:1 molar ratio) was prepared and stirred vigorously at 60 °C. An alkaline solution containing 80 mL of water, 12 g of urea, and 8 g of NaOH was gradually added into the iron solution to form Fe_3_O_4_ nanoparticles. The approximately 8 g of MNPs obtained were washed with water and subsequently with ethanol to remove any nonreactive substances. Then, they were dried in an oven at 60 °C for 48 h, grounded and stored in a desiccator.

The immobilization procedure was adapted from Alsaiari et al.^42^ and Patel et al.^43^ Before functionalization, 2 g of MNPs were dispersed in 400 mL of ethanol/water (1:1 v/v) by sonication for 35 min. Dispersed MNPs were stirred vigorously, and APTES (10% v/v solution) was gradually added to amino-functionalize their surfaces, aiming to provide anchoring points for APTES. Stirring was maintained at 70 °C for 5 h, and then MNPs were centrifuged and washed three times with ethanol/water to remove any free APTES.

Aiming to provide aldehyde groups on MNPs surface for covalently bonding the laccase to the support, glutaraldehyde (2 mol L^–1^) was mixed with the MNPs and 100 mL of sodium-phosphate buffer (pH 7) for 2 h at 24 °C and 150 rpm. The MNPs were centrifuged and washed three times with water to remove any free glutaraldehyde. The enzyme (100 mg of protein per g of support) was immobilized on functionalized MNPs by keeping them in contact for 24 h at 4 °C and 150 rpm in 50 mL of citrate-phosphate buffer (pH 3.5).

After immobilization, the Lac-MNPs were separated by centrifugation (supernatant collected for enzyme assay) and washed three times with citrate-phosphate buffer. The Lac-MNPs were dispersed in citrate-phosphate buffer (pH 3.5) and stored at 4 °C.

### 2.3 Characterization of Free and Immobilized Laccase

Laccase activity was determined by monitoring the oxidation rate of the ABTS substrate. The reaction mixture consisted of 2450 μL citrate-phosphate buffer (pH 3.5), 500 μL ABTS (1.5 mmol L^–1^) and 50 μL of Lac or 10 mg of Lac-MNPs sample. After incubation for 4 min, substrate oxidation was monitored by reading the absorbance at 420 nm (ε_420_= 36,000 L mol^–1^ cm^–1^). The enzyme unit (U) of laccase was defined as the enzyme amount required to oxidize 1 μmol of substrate per minute at 45 °C.^44^ Immobilization efficiency was expressed as residual activity (RA), relative to free enzyme activity (set at 100%).

The activity of Lac-MNPs was monitored during storage and expressed in terms of RA. Leaching tests were performed by mixing the Lac-MNPs with sodium-phosphate buffer (pH 6.5) and keeping them in a shaker at 150 rpm and 24 °C. The supernatant was periodically collected for up to 24 h, and the laccase activity was measured spectrophotometrically.

The activity of free and immobilized laccase at different pH was also investigated by incubating the enzyme for 4 min at 45 °C in citrate-phosphate buffer with a pH range of 2 to 9. Temperature profiles were determined by incubating free and immobilized enzymes at temperatures from 25 to 65 °C at a fixed pH of 3.5.

To evaluate the reusability of the immobilized enzyme, 2 mg of Lac-MNPs were subjected to 10 cycles of laccase activity measurement using the ABTS substrate. After each cycle, the Lac-MNPs were recovered with a magnet and washed to remove any residual ABTS.

### 2.4 Exploring Laccase Resistance

To investigate the laccase resistance to conditions similar to those required for CBZ degradation reactions (see Section 2.5), the enzyme was subjected to (1) UVC irradiation, (2) H_2_O_2_ doses, and (3) ABTS oxidation in continuous flow.

The UVC resistance assays were conducted by exposing both free and immobilized laccase to UV light. The experiments were carried out in a closed cabinet equipped with a disinfectant wand (Crystal Clear-Raze 18 W) emitting radiation at λ_max_ = 254 nm and a fixed irradiance of 42.5 W m^–2^. Samples of 50 μL were periodically collected over 60 min to measure enzyme activity. For H_2_O_2_ resistance testing, 1 mL of free laccase and 1 mL of H_2_O_2_ at varying initial doses (0 to 13.1 mmol L^–1^) were mixed and maintained at room temperature. Samples of 50 μL were collected for enzyme assay after 30 and 60 min of contact time.

Continuous flow ABTS oxidation was performed by placing the laccase extract in a syringe (after measuring the initial activity) and the reaction media (buffer and ABTS) in another syringe. Both were connected to a mixer and precision syringe pumps. The pumps were operated at fixed flow rates corresponding to the same reaction time (4 min) and medium:enzyme ratio (59:1 v/v) as the batch assays.

The inlet solutions were continuously fed to a 3D-printed flat-plate reactor (see Figure 1), with internal flow geometries optimized by Ramos et al.^45^ and detailed in Figure S1. The microdevice has a reaction volume of 5.1 cm^3^ and an irradiated area of 16.7 cm^2^. The flow distribution pattern behaves as plug flow. Using saturated methyl orange aqueous solution as a tracer, the deviation between space-time and residence time was 0.04 min.^45^

**Figure 1:**
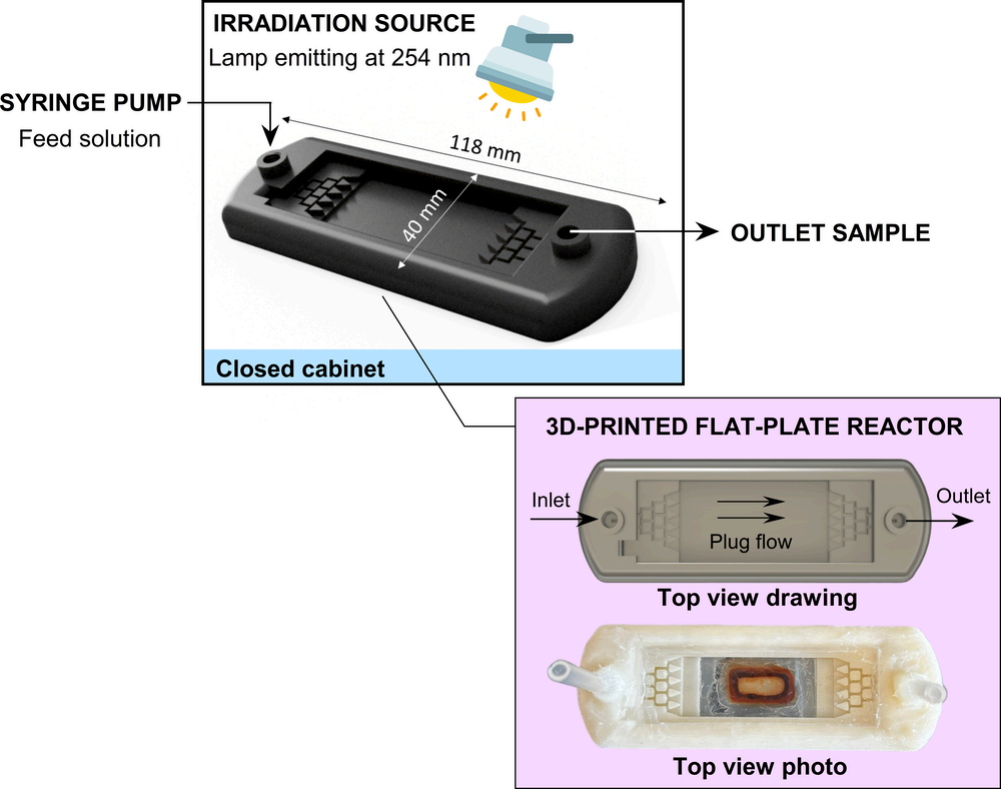
Scheme of the apparatus for the continuous flow experiments with a flat-plate reactor (reaction volume of 5.1 mL). Note: The photograph in the top view shows the Lac-MNPs material (brown content) adhered to a magnet.

Samples of 50 μL were collected from the reactor outlet for absorbance measurement at 420 nm. Enzyme activity (EA) was compared with batch reaction conditions, and the results expressed in terms of relative laccase activity (EA/EA_0_), where EA_0_ is the enzyme activity from batch reaction assays.

### 2.5 Carbamazepine Degradation in Batch Mode

The removal of carbamazepine from contaminated water was evaluated through batch operation in prospective assays. The degradation rate was determined by monitoring the concentration over reaction time. These assays are important for understanding the contribution of each mechanism to carbamazepine removal.

To ensure that CBZ does not decompose due to pH changes, hydrolysis assays were carried out under acidic (pH 4), basic (pH 10) and natural (pH ∼ 7.5) conditions for [CBZ]_0_ = 5 mg L^–1^. Assays were conducted in flasks kept in the dark at 25 °C and shaken at 120 rpm. Samples were collected at 0, 24, and 48 h and analyzed by liquid chromatography.

In addition, the decadic molar absorption coefficient (ε) of CBZ was calculated using the Lambert–Beer Law. Solutions of 1 to 5 mg L^–1^ were scanned between 200 and 800 nm using a UV/vis Varian Cary 50 spectrophotometer and a 1 cm path-length quartz cuvette (see Figure S2). The direct photolysis quantum yield was calculated from the absorptivity data for λ_250–260_, the lamp irradiance, and the degradation experiment by UVC irradiation (direct photolysis).

Batch mode degradation reactions were conducted at a final volume of 40 mL, neutral pH, and [CBZ]_0_ = 5 mg L^–1^. The following batch operation treatment systems were evaluated: (i) Lac-MNPs; (ii) MNPs - adsorption control; (iii) Lac-MNPs/UVC; (iv) MNPs/UVC; (v) UVC - direct photolysis control; (vi) H_2_O_2_, and (vii) UVC/H_2_O_2_. Samples were collected regularly and immediately mixed with methanol to quench the reaction. The pseudo-first order reaction rate constant (*k*) was calculated through linear regression by correlating – ln­([CBZ]/ [CBZ]_0_) values and the reaction time. Specific degradation rates were evaluated to determine system feasibility. The removal efficiency was calculated by eq S1.

Lac-MNPs treatments were carried out at enzyme concentrations of 4 U or 30 U. An additional assay was performed adding [ABTS] = 30 μmol L^–1^ as a laccase inducer. Experiments with MNPs were carried out using 0.8 g L^–1^ of the material. H_2_O_2_ tests were performed with a H_2_O_2_:CBZ molar ratio of 40:1, corresponding to the stoichiometric amount required to achieve complete CBZ mineralization (see eq S2). UVC irradiance was fixed at 42.5 W m^–2^.

### 2.6 Optimizing the Continuous Flow Treatment for Carbamazepine Removal

While ABTS is an excellent laccase mediator for carbamazepine removal,^46^ degradation by chemical reactions is H_2_O_2_-dose-dependent.^47,48^ Hence, the UVC/H_2_O_2_ system under continuous flow regime was initially studied with H_2_O_2_:CBZ molar ratios ranging from 10 to 200. Control experiments were conducted combining MNPs and UVC/H_2_O_2_ to understand the role of photo-Fenton reactions on carbamazepine removal.

Continuous flow assays used the reactor illustrated in Figure 1, with [CBZ]_0_ = 5 mg L^–1^ and a fixed volumetric flow rate corresponding to a space-time of 10 min. Outlet samples were periodically collected until steady-state CBZ concentration was reached. Methanol was used to quench the reaction before CBZ quantification.

To combine laccase and photo-Fenton reactions, the treatment system was optimized following a Central Composite Rotatable Design (CCRD) with eight runs and three replicates under central point conditions. The variables were H_2_O_2_:CBZ molar ratio (*X*_1_, ranging from 2:1 to 23:1) and ABTS concentration (*X*_2_, ranging from 1 to 35 μmol L^–1^). The Lac-MNPs concentration was set at 0.8 g L^–1^ (1.5 U), and the laccase-containing material was distributed within the reaction volume adhered to a NdFeB magnet. For the Lac-MNPs/ABTS/UVC/H_2_O_2_ system, irradiance was set at 42.5 W m^–2^.

### 2.7 Analytical Methods

Surface area and pore analyses were carried out at the Materials Characterization Facility (MCF), Ontario Tech University, using a NOVA 1200e BET nitrogen absorption instrument. The MNPs and Lac-MNPs samples were degassed for 3 h at 200 °C under vacuum conditions, followed by N_2_ gas physisorption analysis using the static volumetric method. The surface area was calculated using the Brunauer–Emmett–Teller (BET) multipoint method. The *P*/*P*_0_ range of >0.15 was chosen for the density functional theory (DFT) modeling method to calculate the average pore volume. The Dubinin-Astakhov (DA) method was used to determine the average pore diameter.

Carbamazepine concentrations were monitored by liquid chromatography-tandem mass spectrometry (LCMS/MS) in positive ionization mode using a Shimadzu HPLC (SIL-40CXR) and Sciex MS (QTRAP 5500). Transformation products (TPs) from CBZ degradation by the Lac-MNPs/ABTS/UVC/H_2_O_2_ continuous flow system were determined using a Sciex QTOF MS/MS (ZenoTOF 7600 System) equipped with an electrospray ionization source (ESI) in positive ionization mode. Operational details are provided in the supporting material (Table S1).

### 2.8 Ecotoxicity Prediction to Aquatic Organisms

The ecotoxicity of CBZ and the TPs to fish, daphnids, and green algae was estimated using ECOSAR software version 2.2 (U.S. Environmental Protection Agency). The software predicts acute and chronic toxicity to the aquatic organisms using Quantitative Structure Activity Relationships (QSARs). The values were used to calculate the predicted no-effect concentration (PNEC) by dividing the lowest predicted concentration (EC_50_ or LC_50_) by an uncertainty factor of 1000. The concentration of concern (COC) was also calculated by dividing the lowest chronic concentration (ChV) by an uncertainty factor of 10.^49,50^

### 2.9 Statistical Analysis

The experiments were carried out in triplicate, and the data were analyzed using analysis of variance (ANOVA) and Tukey test at a p-value <0.05 using Statistica 8.0 software and Protimiza Experimental Design available online at http://experimental-design.protimiza.com.br/.^51^

## 3 Results and Discussion

### 3.1 Characteristics of Free and Immobilized Laccase on Magnetite Nanoparticles

Laccase was effectively bound to MNPs surfaces, resulting in 1.87 ± 0.08 U per mg of support (U mg^–1^). During Lac-MNPs washing procedure, a theoretical loss of 25% of enzyme was obtained, as indicated by measuring the activity of the supernatant. Leaching tests showed no enzyme loss over 24 h, indicating a strong bond between the enzyme and the surface of the support. As the MNPs were functionalized before immobilization, the amino and aldehyde groups on their surfaces could be responsible for multipoint interaction with laccase.^52,53^

The Lac-MNPs material was stored in a refrigerator, dispersed in citrate-phosphate buffer, and Lac activity was measured periodically. As shown in Figure 2a, the Lac-MNPs presented good storage stability, with activity of 1.35 ± 0.25 U mg^–1^ on day 14, representing a 28% reduction compared to day 0. Between days 21 and 50, the activity remained stable at approximately 0.96 U mg^–1^, indicating a 49% reduction compared to day 0. However, by day 77, the activity decreased to 0.34 U mg^–1^. Overall, Lac immobilized on MNPs exhibited good immobilization efficiency and promising storage stability.

**Figure 2:**
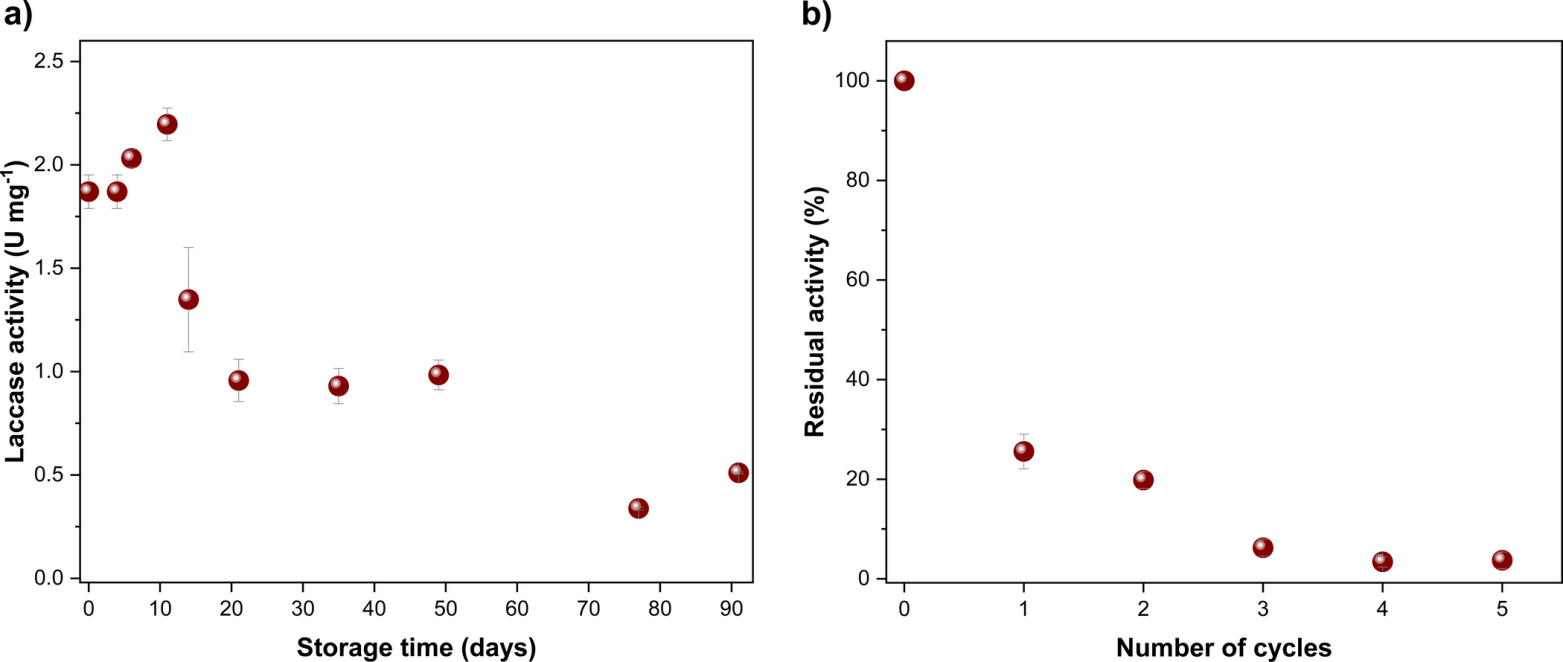
Activity of the Lac-MNPs material during (a) storage at 4 °C in citrate-phosphate buffer, and (b) reuse cycles for ABTS oxidation.

Regarding reusability for ABTS oxidation, the Lac-MNPs residual activity was 25.6 ± 3.5% in the first cycle, dropping to 3.7 ± 1.0% in the fifth cycle (see Figure 2b). From cycles 6 to 10, the activity of the reused Lac-MNPs material was zero. The stable enzyme-support bond revealed by the leaching tests suggest that the rapid decrease in enzyme activity can be attributed to catalytic site saturation or protein denaturation, as reported in other studies.^42,54^

Physical properties of magnetite nanoparticles with and without immobilized enzyme are compared in Table 1. The results indicate reduced surface area and pore volume for the Lac-MNPs material. This outcome was expected, as the immobilization method involved chemically bonding the enzyme to the surface of the MNPs. The pore diameters of both MNPs and Lac-MNPs were similar (2.14 and 3.18 nm, respectively) and can be classified as falling within the range of micro and mesoporous structures, according to IUPAC guidelines.

**Table 1:** Surface Area and Pore Characteristics of Fe_3_O_4_ Nanoparticles (MNPs) and Lac-Containing Material (Lac-MNPs)^a^

**Sample**	**BET surface area (m** ^**2**^ **g** ^**–1**^ **)**	**DFT pore volume (cm** ^**3**^ **g** ^**–1**^ **)**	**DA pore diameter (nm)**
MNPs	177.6	0.15	2.14
Lac-MNPs	18.8	0.04	3.18

a BET: Brunauer–Emmett–Teller multipoint method; DFT: Density Functional Theory modeling method; DA: Dubinin-Astakhov method.

During the immobilization process, in addition to Lac adhering to the surfaces of MNPs, enzyme aggregation and conformational changes within the support material are likely to occur,^55,56^ resulting in decreased surface area. Compared to commercial magnetite, which has a surface area between 20 and 50 m^2^ g^–1^,^26^ the Fe_3_O_4_ nanoparticles synthesized in this work have a superior surface area, which allows higher enzyme loading.^55^

The effect of pH during incubation for both free and immobilized Lac showed similar behavior, as illustrated in Figure 3a. Higher activities were observed in acidic media (pH 2 to 4), with a decline to zero in neutral and alkaline media (pH 6 to 9). As depicted in Figure 3b, free laccase showed higher activity at 45 and 55 °C, while the immobilized enzyme remained stable across the studied range, maintaining a residual activity above 60%. Notably, the immobilized enzyme exhibited a higher residual activity (84 ± 4%) compared to its free form (49 ± 7%) at 25 °C, which is significant given that the treatment system for carbamazepine removal was evaluated at this temperature.

**Figure 3:**
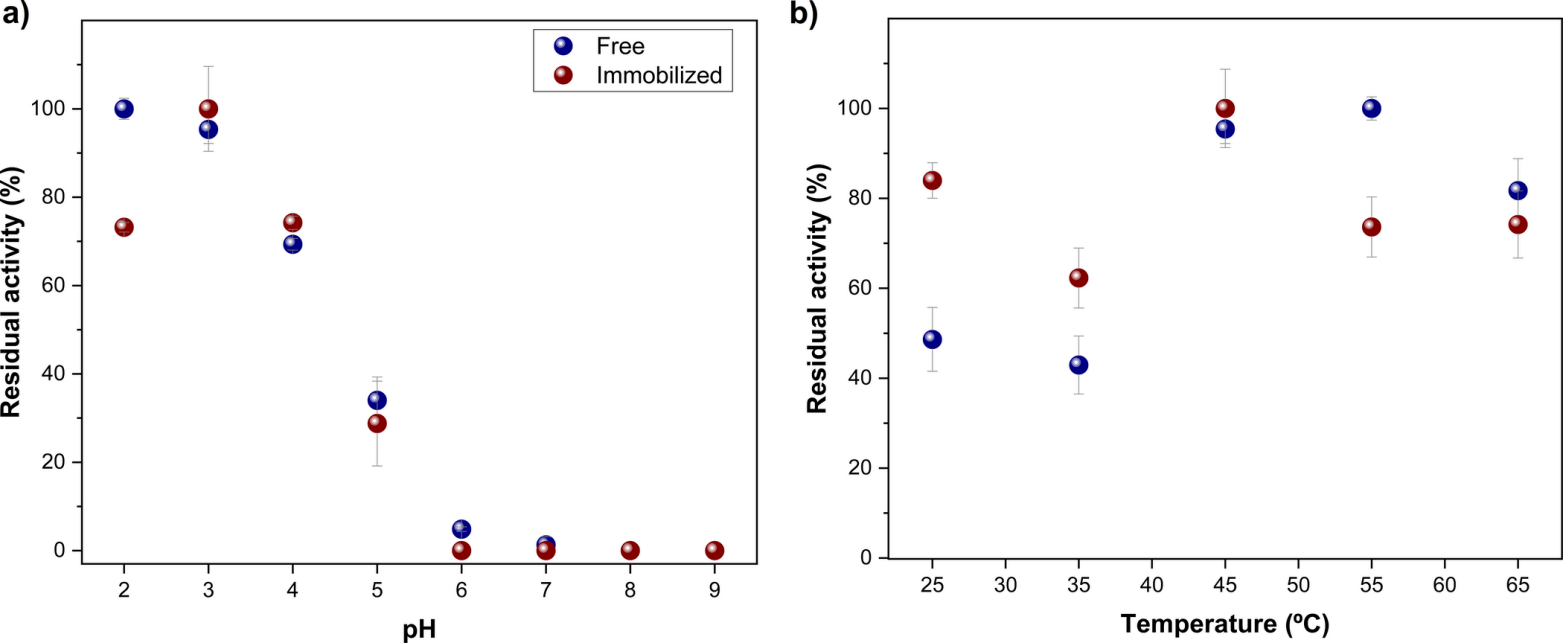
Activity of free (blue symbols) and immobilized (burgundy symbols) laccase at different (a) pH and (b) temperatures.

### 3.2 Laccase Resistance under Treatment Conditions

As the proposed treatment approach is based on using the enzyme with H_2_O_2_ under UVC irradiation in a single reactor, the stability of free and immobilized laccase from *Trametes versicolor* was tested under these conditions. From the initial activity, set as 100%, free enzyme activity decreased to 36.4 ± 4.6% after 5 min and 5.7 ± 1.9% after 15 min of UVC exposure, exhibiting an exponential decay and reaching zero after 30 min (Figure S3). In contrast, the immobilized enzyme retained 77.7 ± 5.5% activity after 60 min. Lac-MNPs showed superior resistance to UVC exposure, suggesting that bonding the enzyme to magnetite protects against protein damage and provides stability, reinforcing the operational advantages of immobilizing enzymes.^57,58^

After 30 and 60 min of contact with H_2_O_2_ doses ranging from 0 to 13.1 mmol L^–1^, laccase activity remained statistically unchanged, according to the Tukey test (p-value <0.05). The resistance of *T. versicolor* laccase to UVC radiation and H_2_O_2_ had not yet been reported in the literature, although spore-associated laccases are known to protect microorganisms from such external agents.^59,60^ In turn, oxidoreductase enzymes such as chlorite dismutase and horseradish peroxidase were found to be photosensitive under visible light (∼400 nm) due to heme group degradation, conformational changes, and protein precipitation.^61^

Finally, to assess ABTS oxidation by laccase under continuous flow, the activity of free Lac was set at EA_0_ = 1.0 (batch assay). The relative activity under continuous flow, at a space-time of 4 min, reached 1.08 ± 0.08 after 15 min of reactor operation and stabilized at 1.34 ± 0.01 after 30 min (Figure S4). Relative activity values exceeding 1.0 indicate that ABTS oxidation by laccase is more effective in continuous flow regime compared to batch mode. This promising result shows the affinity of laccase for this type of microdevice. However, using free enzyme is unsuitable due to the low stability under UVC exposure and degradation issues discussed in Section 3.3. Nevertheless, these findings highlight the excellent performance of laccase in biocatalytic reactions under continuous flow operation, making it valuable for researchers developing biosensors for detecting molecules oxidized by laccase, which is a trending topic in areas such as environmental monitoring, food safety, and drug formulations.^62,63^

### 3.3 Batch Mode Removal Performance

Hydrolysis experiments indicated that CBZ degradation due to pH changes or thermal decomposition is negligible. Figure S2 shows the CBZ molar absorption coefficient for 200–800 nm, suggesting susceptibility to direct photolysis at wavelengths below 320 nm. Although the ε value at 254 nm (UVC wavelength) is high (7340 L mol^–1^ cm^–1^) and consistent with other studies,^25^ a low quantum yield (Φ_UVC_ = 3.04 × 10^–4^ mol Einstein^–1^) was obtained from the direct photolysis experiment (UVC treatment, Figure 4).

**Figure 4:**
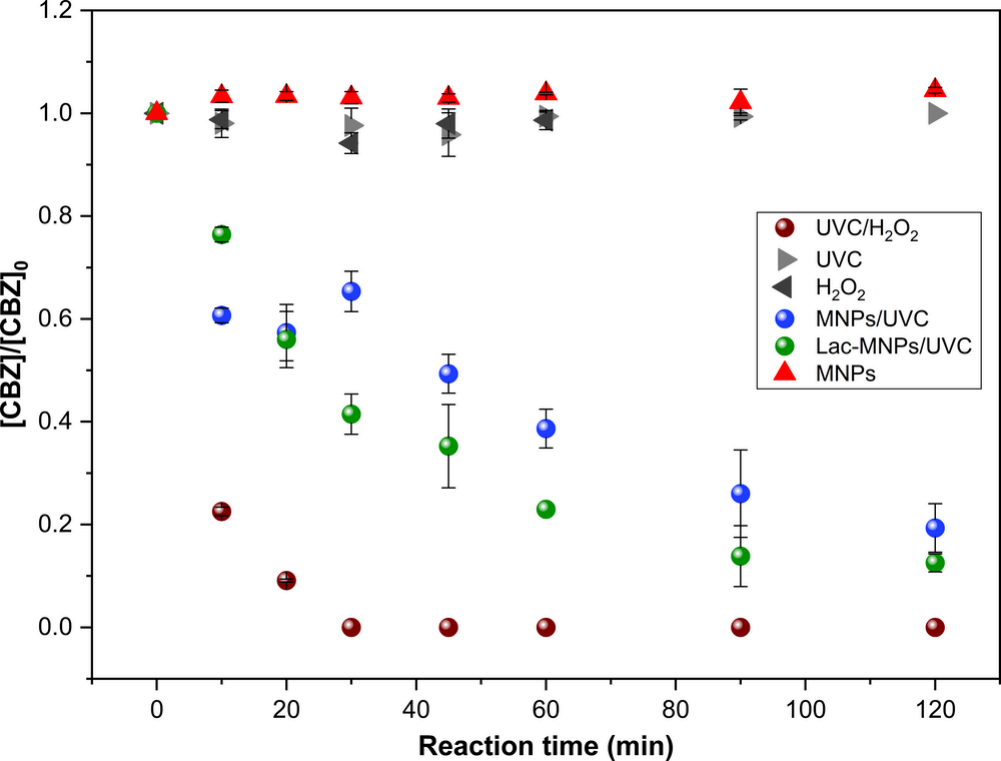
Results of batch mode experiments ([CBZ]_0_ = 5.01 ± 0.73 mg L^–1^) for carbamazepine removal.

In addition, CBZ was not degraded by H_2_O_2_ alone at a 40:1 H_2_O_2_:CBZ molar ratio (stoichiometric amount). Similarly, Gabet et al.^26^ observed that CBZ remained stable against pH changes (pH 3.5 or 7.5), H_2_O_2_ (100:1 H_2_O_2_:CBZ molar ratio), and UVA radiation (λ_max_ = 352 nm). Even combining magnetite with UVA and citrate, the authors observed less than 10% CBZ removal after 3 h, reinforcing CBZ persistence.

The adsorption control assay with only MNPs showed no CBZ removal (Figure 4). In contrast, the UVC/H_2_O_2_ process achieved a *k* value of 0.125 ± 0.002 min^–1^ (R^2^ = 0.998) and 100% removal in 1 h of reaction at stoichiometric amount (Table 2). Odabasi and Buyukgungor^64^ reported the high removal efficiency of the UVC/H_2_O_2_ process using 0.14 mmol L^–1^ of H_2_O_2_, with complete CBZ removal after 30 min. However, lower concentrations of H_2_O_2_ resulted in decreased efficiency, with approximately 40% removal at 0.035 mmol L^–1^ of H_2_O_2_. This finding illustrates the dose-dependent behavior of CBZ removal by this process, whereby the main degradation mechanism is based on hydroxyl radicals attacking the pollutant through the photolysis of hydrogen peroxide.^23^

**Table 2:** Percent Removals and Pseudo-first Order Specific Degradation Rates (*k*) of Carbamazepine in Batch Treatments with 30 U Enzyme Concentration and 30 μmol L^–1^ ABTS

**Assay**	**Removal in 1 h (%)**	***k* (min^–1^)**	**R** **^2^**
Lac-MNPs/UVC	77.0 ± 0.1^a^	0.022 ± 0.001^a^	0.973
MNPs/UVC	61.3 ± 3.8^b^	0.015 ± 0.001^a^	0.962
Lac-MNPs/ABTS/UVC/H_2_O_2_ [Table-fn t2fn1]	99.4 ± 0.2^c^	0.146 ± 0.008^b^	0.994
UVC/H_2_O_2_ [Table-fn t2fn2]	100^c^	0.125 ± 0.002^c^	0.998

A H_2_O_2_:CBZ molar ratio at 20:1;

B H_2_O_2_:CBZ molar ratio at 40:1. Equal lowercase letters indicate that the samples do not differ by the Tukey test (p-value <0.05).

The results also indicate that CBZ does not undergo degradation solely due to the action of Lac-MNPs when used at concentrations of 4 U and 30 U after 2 h of reaction. Previous studies have reported that both free and immobilized laccase achieved remarkable removal results (≥80%) after 24 h of batch reaction. In contrast, for a 3-h reaction period, free laccase achieved less than 10% removal, irrespective of the use ABTS as an inducer.^46,65^

The treatment system for CBZ removal combining laccase and ABTS was optimized by Naghdi et al.^46^ following a Central Composite Design, and at the optimal condition (35 °C, 18 μmol L^–1^ ABTS, pH 6, and 60 U L^–1^ of Lac) the treatment required 24 h of batch reaction to achieve 94.75% CBZ removal. The authors also compared the system with and without the mediator and observed superior performance of Lac-ABTS treatment compared to Lac treatment from 3 to 24 h of batch reaction. This improvement was attributed to the ability of oxidized ABTS to generate reactive radicals and to stabilize and protect the enzyme during the reaction.^46^ The combined reactions of laccase and ABTS in CBZ degradation are shown in Figure 5. During the biocatalytic cycle, laccase oxidizes ABTS to the ABTS cation radical, which subsequently acts as a redox mediator to oxidize CBZ, generating CBZ cation radicals and others intermediate products.^66^

**Figure 5:**
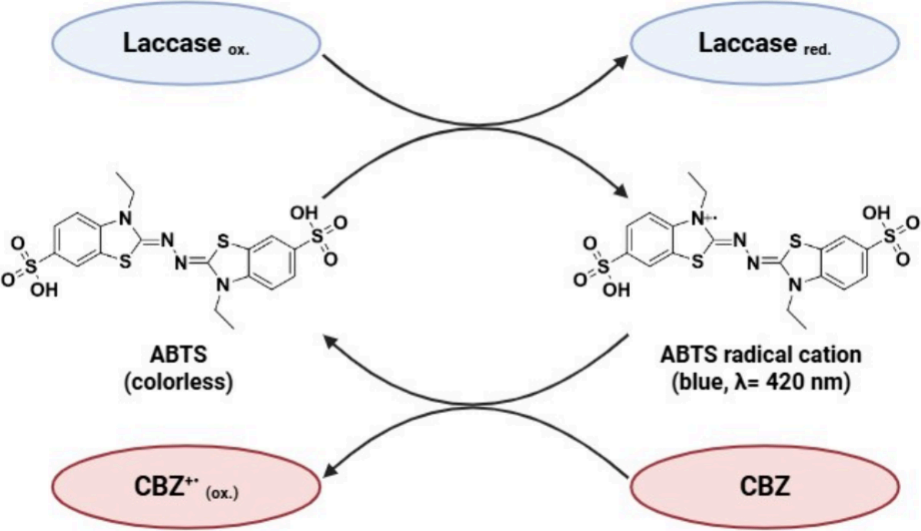
Reaction mechanisms of laccase-ABTS system for carbamazepine degradation.

Compared with the UVC/H_2_O_2_ process, using the enzyme alone or the Lac-ABTS system is not considered time-effective given the low degradation rate of enzymatic reaction. Improved results were obtained with the Lac-MNPs/UVC system, which achieved 77.0 ± 0.1% removal in 60 min. As shown in Table 2, the Lac-MNPs/UVC system demonstrated superior CBZ removal compared to MNPs/UVC (61.3 ± 3.8%), confirmed by the Tukey test (p-value <0.05), indicating that the presence of the enzyme enhances system performance.

The CBZ removal by MNPs/UVC treatment is due to photodegradation, with magnetite nanoparticles acting as a photocatalyst.^67–69^ As shown in Figure 4, Lac-MNPs/UVC performs better than MNPs/UVC alone, suggesting a synergistic effect of enzymatic-photocatalysis for CBZ degradation. Notably, H_2_O_2_ was not added to these two systems; thus, UVC/H_2_O_2_ or Fenton-like reactions were not induced.

Considering the conditioned efficiency of the conventional UVC/H_2_O_2_ process to the addition of high H_2_O_2_ concentrations (≥28.8 mg L^–1^), and the lower degradation rate of Lac-MNPs/UVC system (*k* = 0.022 ± 0.001 min^–1^), a single-step treatment was evaluated, resulting in 99.4 ± 0.2% removal in 60 min (*k* = 0.146 ± 0.008 min^–1^). The Lac-MNPs/ABTS/UVC/H_2_O_2_ treatment matched the removal results of the UVC/H_2_O_2_ system (as confirmed by the Tukey test, Table 2), but with half the H_2_O_2_ dose (20:1 H_2_O_2_:CBZ molar ratio, equivalent to 14.4 mg L^–1^). The *k* value for the Lac-MNPs/ABTS/UVC/H_2_O_2_ treatment was statistically superior according to the Tukey test.

In addition to the economic benefits of reducing the amount of hydrogen peroxide used in the reaction, minimizing chemicals usage also enhances the environmental safety of the process. Adding too much oxidant can leave residual H_2_O_2_ in wastewater, causing environmental hazards due to its toxicity. For example, acute toxicity results of H_2_O_2_, estimated using ECOSAR software, have shown an effective concentration (EC_50_) of 1.62 mg L^–1^ for green algae and a lethal concentration (LC_50_) of 3.30 mg L^–1^ for fish.

As shown in Figure 5, the enzyme and its mediator act together to generate reactive radicals that attack the CBZ molecule. Similarly, when combining MNPs, UVC, and H_2_O_2_, combined reactions occur in the treatment system. This combination is known as the photo-Fenton process, in which MNPs containing ferrous iron react with H_2_O_2_ under UVC irradiation. The light source (*hv*) also interacts with the MNPs. Concurrent reactions are important for generating additional •OH radicals compared to other advanced oxidation processes.^70–72^ These potent oxidizing species are continuously generated during the photo-Fenton process and are responsible for attacking the CBZ molecule and transforming it into oxidized byproducts.^23,71,73^ The photo-Fenton reactions are presented in eq 1–8.

$${\mathrm{Fe}}^{3+}+H_{2}O_{2}+hv\to {\mathrm{Fe}}^{2+}+{\mathrm{HO}}_{2}^{•}+H^{+}$$ 1

Fe3++H2O+hv→Fe2++OH•+H+ 2

Fe2++H2O2→Fe3++OH−+OH• 3

Fe2++OH•→Fe3++OH− 4

H2O2+hv→2OH• 5

H2O2+OH•→H2O+HO2• 6

OH•+OH•→H2O2 7

CBZ+OH•→transformationproducts 8

### 3.4 Treatment System Optimization under a Continuous Flow Regime

The UVC/H_2_O_2_ system was studied for various H_2_O_2_:CBZ molar ratios under continuous flow, alongside the MNPs/UVC/H_2_O_2_ system (photo-Fenton reaction). At a fixed [H_2_O_2_]_0_ of 7.2 mg L^–1^ (10:1 molar ratio), the photo-Fenton process using MNPs achieved 59.4 ± 2.6% removal, which was lower compared to the UVC/H_2_O_2_ system (74.1 ± 1.1% removal) for the same inlet hydrogen peroxide dose.

For higher doses of H_2_O_2_ (≥28.8 mg L^–1^), the UVC/H_2_O_2_ process achieved ≥ 97% CBZ removal. Giannakis et al.^74^ explained that UVC/H_2_O_2_ reactions produce more hydroxyl radicals than the photo-Fenton process at neutral pH. These radicals are responsible for attacking pollutant molecules and increasing their degradation. One of the limiting factors for photo-Fenton reactions is that the reduction of H_2_O_2_ into hydroxyl radicals depends on H_2_O_2_ adsorption onto MNPs surfaces.^75^

As shown in Table 3, the hybrid treatment (Lac-MNPs/ABTS/UVC/H_2_O_2_ system) with [H_2_O_2_]_0_ = 3.6 mg L^–1^ (5:1 molar ratio) and [ABTS]_0_ = 6 μmol L^–1^ (CCRD experiment 1, Table 3) resulted in 59.1% CBZ removal. Thus, the performance of the laccase-photo-Fenton system was superior to that of conventional photo-Fenton, given the reduced amount of hydrogen peroxide used for equivalent removal performance. In this sense, aiming to optimize the combined laccase and photo-Fenton reactions in a single-step process while using minimal reactants, the hybrid system was optimized following the CCRD matrix shown in Table 3. The hypothesis for the variables chosen was that higher ABTS concentrations favor laccase reactions, while higher H_2_O_2_ concentrations benefit photo-Fenton reactions. Balancing both degradation mechanisms can improve CBZ removal at lower chemical concentrations, as in excess, they can negatively impact operational efficiency.^23,76^

**Table 3:** CCRD Matrix with Real and Coded Values and the Experimental CBZ Removals

**Experiment**	**Variables**		**Response**
	*X*_1_ – H_2_O_2_:CBZ molar ratio	*X*_2_ – ABTS concentration (μmol L^–1^)	*Y*_1_ – CBZ removal (%)
1	5.0:1.0 (−1)	6 (−1)	59.1
2	20.0:1.0 (+1)	6 (−1)	83.4
3	5.0:1.0 (−1)	30 (+1)	62.8
4	20.0:1.0 (+1)	30 (+1)	91.1
5	1.9:1.0 (−1.41)	18 (0)	32.4
6	23.1:1.0 (+1.41)	18 (0)	83.1
7	12.5:1.0 (0)	1 (−1.41)	67.7
8	12.5:1.0 (0)	35 (+1.41)	66.6
9–11	12.5:1.0 (0)	18 (0)	84.1 ± 4.8

The response surface revealed that experiment 4, with a 20:1 H_2_O_2_:CBZ molar ratio and 30 μmol L^–1^ of ABTS, achieved maximum removal (see Figure 6). Note that the optimal H_2_O_2_ dose corresponds to a substoichiometric amount, a condition in which the UVC/H_2_O_2_ system underperformed. The model was validated by analysis of variance, with R^2^ = 0.8707 and a p-value of 0.028. The empirical mathematical model presented in Table 4 describes the CBZ removal performance by the proposed laccase-photo-Fenton treatment.

**Figure 6:**
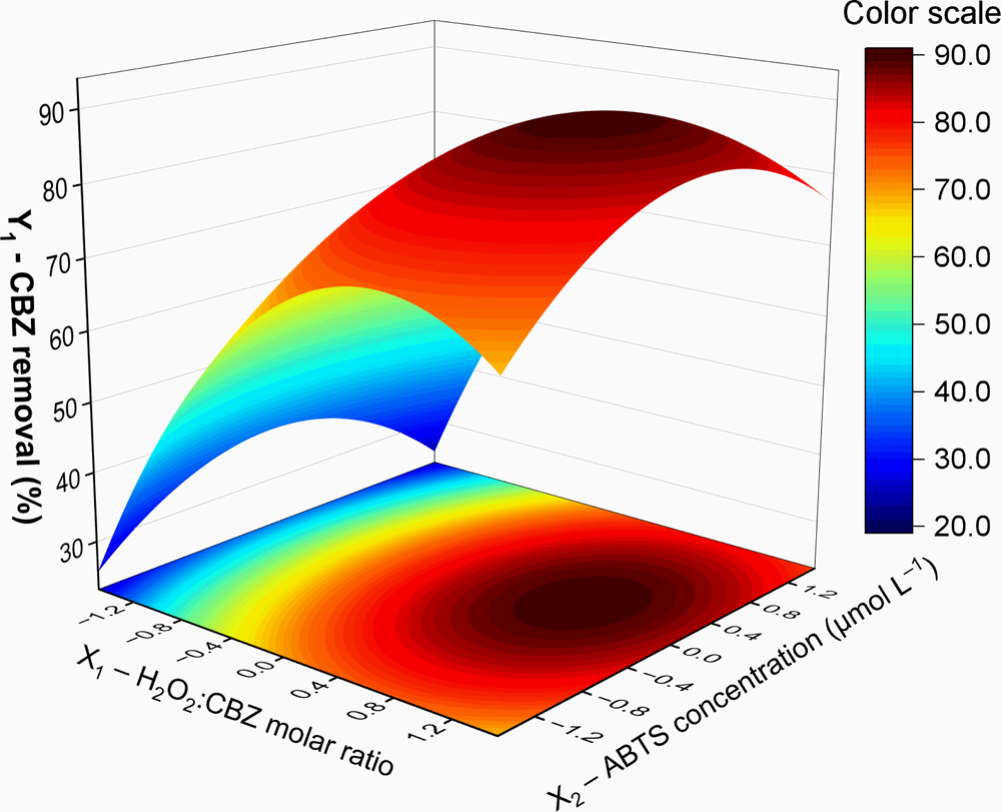
Response surface for the hybrid laccase-photo-Fenton system in continuous flow regime.

**Table 4:** Regression Results for the Continuous-Flow Hybrid System (CCRD Experiments)

**Variation Source**	**Sum of Squares**	**Degrees of Freedom**	**Mean Square**	**p-value**
Regression	2585.96	5	517.19	0.028
Residuals	383.96	5	76.79	
Lack of Fit	338.37	3	112.79	0.173
Pure Error	45.58	2	22.79	
Total	2969.91	10		
Empirical mathematical model	*Y*_1_ = 15.555*X*_1_ – 10.262*X*_1_^2^ + 1.218*X*_2_ – 5.562*X*_2_^2^ + 0.977*X*_1_ *X*_2_ + 84.090			

As a result of monitoring the CBZ concentration during reactor start-up, the laccase-photo-Fenton treatment reached a steady-state regime within 15 min of reactor operation and remained stable for up to 60 min. This is a promising result, representing a viable reactor start-up compared to treatment systems with microorganisms, which require several weeks to reach the steady-state regime.^77,78^

In addition to the optimized experimental condition, it is possible to predict from the mathematical model that a 90% CBZ removal can be achieved at a 17:1 molar ratio with [ABTS]_0_ ranging from 17 to 23 μmol L^–1^. Similarly, the molar ratio can vary from 17 to 19.4 when using [ABTS]_0_ = 17 μmol L^–1^. The operating range for the studied variables provides robustness and reliability to the proposed system, as variations and uncertainties can occur during the operation of a treatment process.

Two advantages of the proposed hybrid system include: (i) reduced amount of H_2_O_2_ compared to the amount required for UVC/H_2_O_2_ reactions and (ii) conduction the treatment at near-neutral pH, which is not common for photo-Fenton reactions as pH ∼ 3 is preferred.^79^ In addition, by comparing the result of the optimum condition (experiment 4, Table 3) for the system operated in continuous flow mode with the same treatment conditions operated in batch mode (Table 2), it can be stated that the proposed treatment performs similarly, regardless of the operation mode. While the batch system achieved 91.9 ± 2.1% removal after 15 min of reaction, the continuous system achieved 91.1% removal operated at a space-time of 10 min. These results demonstrate the versatility and robustness of the proposed treatment system for CBZ removal.

Nevertheless, the continuous flow mode has several advantages over the batch mode, such as (i) continuous reuse of the heterogeneous biocatalysts, which are adhered to a magnet during the process; (ii) uniform illumination of the reaction volume, optimizing light use; (iii) easy operational automation of chemicals input; and (iv) alignment with industrial and social demands, as wastewaters are continuously generated.^80,81^ According to Holtze and Boehling,^80^ flow-processing offers sustainability and economic benefits, but faces high development barriers due to insufficient infrastructure, equipment, expert knowledge, best practices, and a lack of smooth R&D workflow.

In the broader scenario, hybrid approaches that combine biochemical and photochemical reactions to enhance the degradation of organic pollutants are often limited to sequential batch or semibatch reactors, rather than systems that integrate both processes in a single device. Such systems can achieve interesting removal efficiencies, but reaction times typically range from hours to days.^82–87^ In contrast, the hybrid treatment configuration proposed in this work achieved removals of over 90% in both batch and continuous operation modes, using reaction times of only a few minutes. By coupling enzymatic and photo-Fenton processes in a single reactor, outstanding performance was achieved due to the simultaneous generation of reactive radicals from both reaction pathways.

### 3.5 Proposed Degradation Pathway and Byproducts Ecotoxicity

To propose the CBZ degradation pathway for the continuous-flow hybrid system, transformation products were identified using LCMS/MS, as shown in Figure 7. The TPs detected at *m*/*z* 267 (TP1), *m*/*z* 271 (TP2) and *m*/*z* 253 (TP3 and TP4) indicate UV- or hydroxyl-radical-mediated oxidation (including both epoxidation and hydroxylation) through the double bond in the central seven-membered ring of the drug molecule.^88^ The formation of an epoxy-derivative of CBZ (TP3 at *m*/*z* 253) can also result from the dehydration (loss of H_2_O) of the double hydroxylated product of CBZ (CBZ-10,11-diol, TP2 at *m*/*z* = 271), as suggested by a previous study.^48^ The attack of ^•^OH radicals on CBZ-10,11-diol could also produce the intermediate identified at *m*/*z* 287 (TP7), which serves as a precursor for TP14 and TP15 at *m*/*z* 224.^47^

**Figure 7:**
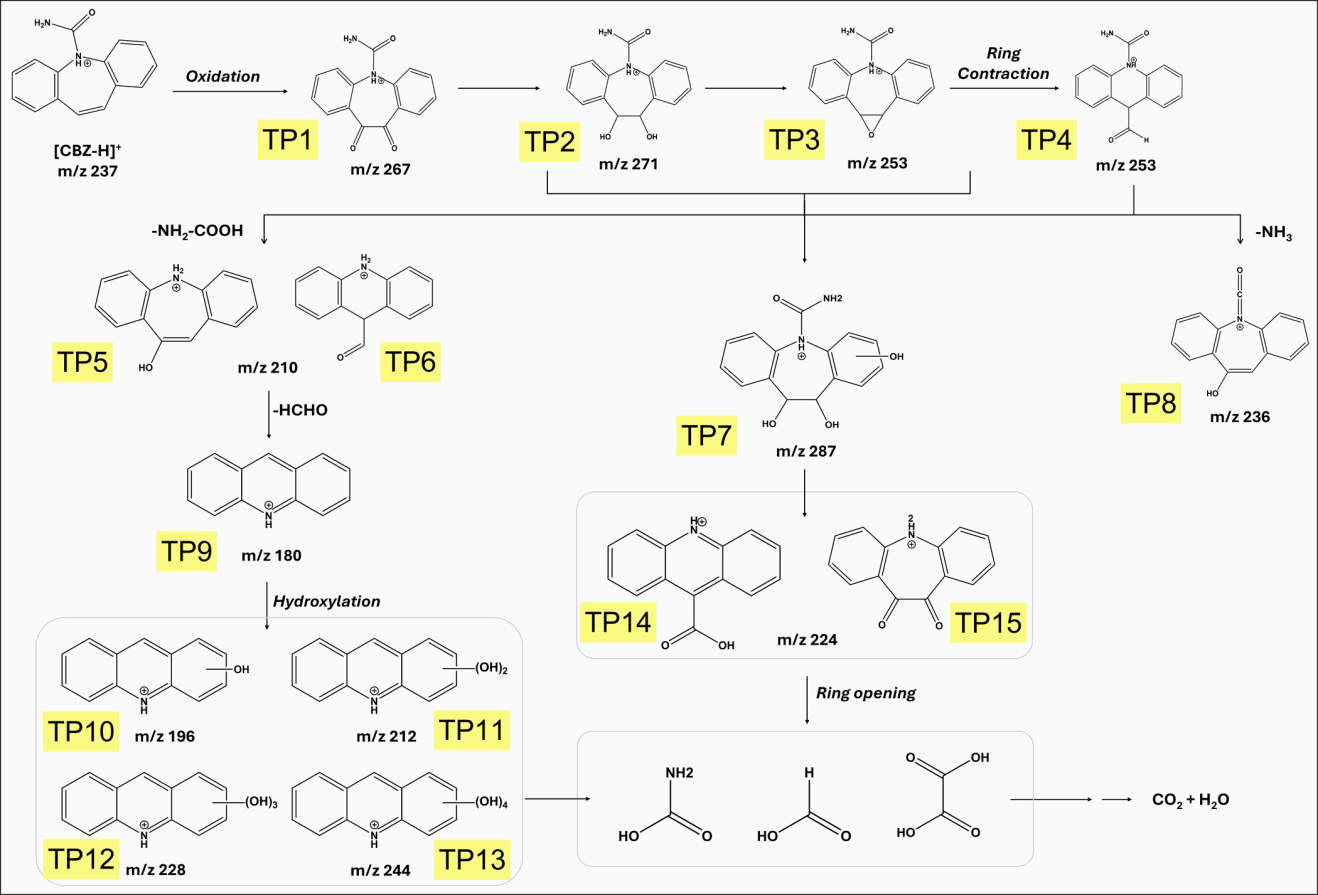
Proposed degradation pathway for CBZ by the hybrid laccase-photo-Fenton system in continuous flow regime.

TP3 and TP4 (*m*/*z* 253) can also lead to the formation of acridine (TP9, *m*/*z* 180) through amine cleavage, hydrogen abstraction, and decarboxylation.^88^ Further, the intermediates undergo ^•^OH mediated chemical reactions that result in ring-opening and formation of lower-molecular-weight organic acids. The observed change in pH of the solution after treatment (from >7 to around 4) further supports the formation of acids following laccase-photo-Fenton treatment.^47^

The physicochemical properties and aquatic toxicity data of the identified TPs were assessed using an *in silico* approach (see Table S2 for the complete data set). From these data, PNEC and COC values were calculated as ecotoxicity indicators, and the compounds were ranked from highest (red bars) to lowest (green bars) concern, as presented in Figure 8. The gray bar represents the data for CBZ (PNEC = 0.055 mg L^–1^ and COC = 0.701 mg L^–1^), which serves as a reference to determine whether the proposed treatment generates more or less harmful substances. For some of the TPs, it was not possible to calculate the indicators as the compounds have no effect at saturation, reported when the effect level exceeds their water solubility.

**Figure 8:**
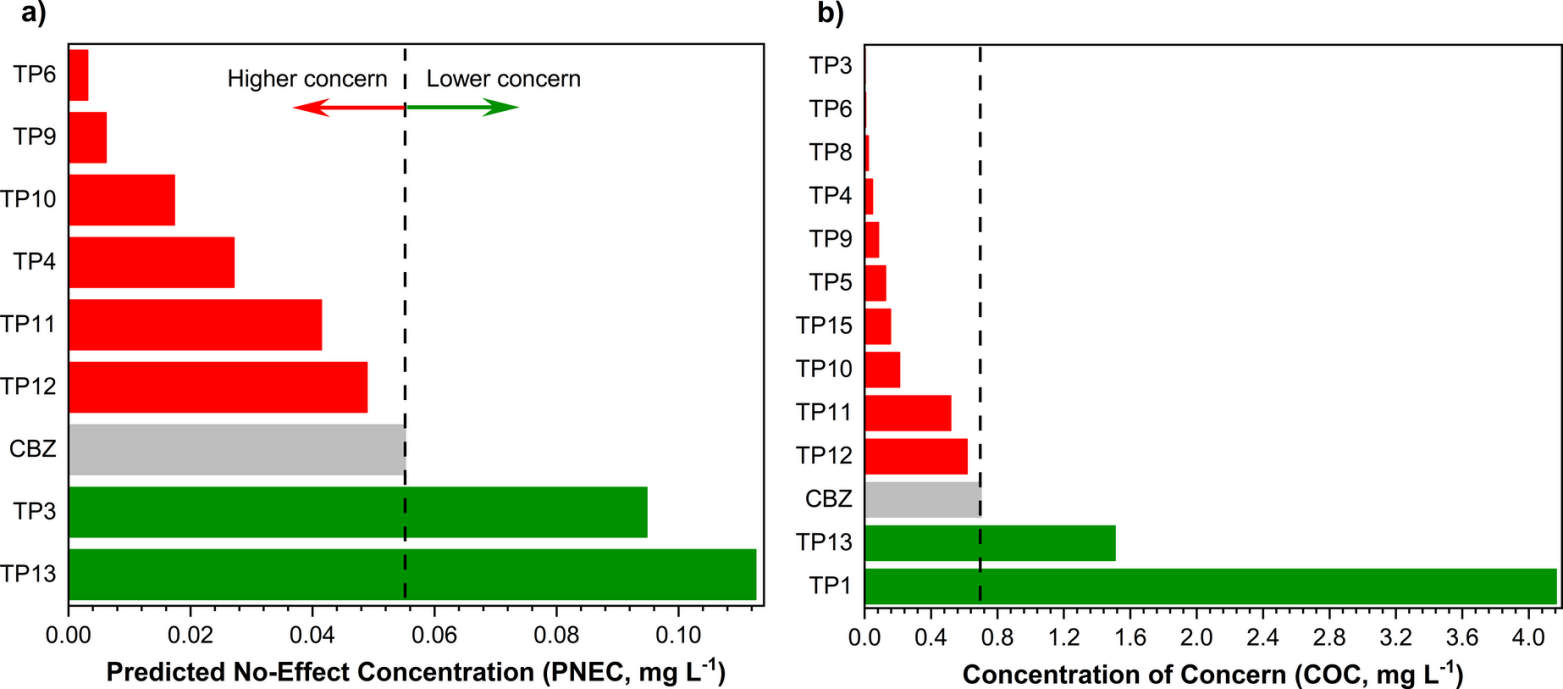
Predicted ecotoxicity indicators based on (a) acute values and (b) chronic values for CBZ and the TPs generated during the degradation process. See Table S2 for the complete data set.

Among the 15 transformation products generated by the laccase-photo-Fenton treatment, only TP13 was considered to pose a lower concern for both acute (PNEC = 0.113 mg L^–1^) and chronic (COC = 1.509 mg L^–1^) toxicity compared to carbamazepine. In addition, the acute risk of TP3 (PNEC = 0.095 mg L^–1^) and chronic risk of TP1 (COC = 4.166 mg L^–1^) are anticipated to present lower concern compared to CBZ.

The compounds of higher concern regarding acute toxicity are TP6 and TP9, and TP3, TP6, TP8, and TP4 for chronic toxicity. These compounds exhibited PNEC and/or COC values up to 10 times lower than the parent compound. Studies by Ali et al.^48^ and Trognon et al.^89^ indicated that AOP-based treatments can generate harmful substances but emphasized the need to optimize technologies for removing CBZ from water and develop analytical tools to quantify TP concentrations.

Carbamazepine-10,11-epoxide (TP3 and TP4, *m*/*z* 253) is a well-known metabolite of carbamazepine, and its toxicological effects to zebrafish embryos were assessed by Rodrigues et al.^90^ Compared to CBZ, the carbamazepine-10,11-epoxide increased the embryonic malformation rates and the authors alert to the significant environmental risks associated with releasing this metabolite in aquatic systems. It is worth noting that carbamazepine-10,11-epoxide occurrence in water matrices is also associated with carbamazepine metabolization by the human liver, which generates this compound as main metabolite.^90–92^

Acridine 9-carboxylic acid (TP14 and TP15, *m*/*z* 224) was studied by Desbiolles et al.^93^ using two species from different trophic levels. The plant bioassay using *Lemna minor* L. for 17 days of exposure resulted in a significantly increased nitrogen balance index compared to the parent compound, while no significant differences were detected for the index of phenolic compounds and chlorophylls. The animal bioassay using *Hydra circumcincta* resulted in no alteration of the feeding behavior during the 2 weeks of exposure to CBZ or acridine 9-carboxylic acid, while both compounds caused oxidative stress. The authors highlight that no specific trend could be identified because complex interactions occur between the compound and the metabolism of the tested organisms.

Finally, acridine (TP9, *m*/*z* 180) and acridone (TP10, *m*/*z* 196) were studied by Donner et al.^94^ following the International Standard Organization (ISO) protocols. The model organisms were the bacteria *Vibrio fischeri*, the algae *Pseudokirchneriella subcapitata*, and the crustacean *Daphnia magna*. The exposure to both TPs resulted in superior acute toxicity to the three organisms compared to the parent compound. Moreover, TP10 resulted in lower toxicity than TP9 for bacterial inhibition of bioluminescence, algal inhibition of growth, and *Daphnia* immobilization, as also predicted using the *in silico* approach (see Figure 8). By comparing the *in vivo* results and the predicted ecotoxicity, it becomes evident the acute and chronic risks of the byproducts generated during carbamazepine degradation.

The ecotoxicity parameters estimated using quantitative structure–activity relationships models have been recognized by the regulatory agencies as a useful screening and predictive tool to support risk prioritization and risk assessment frameworks.^95^ Since the effects of a specific substance on living organisms depend on several factors, including routes and patterns of exposure, concentration in water and environmental parameters,^96^ it is not possible to definitively state whether the post-treatment solution is more or less toxic than the solution containing only CBZ. It is worth noting that if not quenched, the free radicals responsible for hydroxylation and ring opening continue to act in TPs degradation, forming small organic acids and leading to the mineralization of CBZ, as proposed in Figure 7.

## 4 Conclusions

For the first time, a hybrid laccase-photo-Fenton treatment for effective carbamazepine removal in both batch and continuous flow regimes at room temperature and neutral pH was investigated. To enhance the stability and activity of laccase, the enzyme was immobilized on functionalized magnetic iron oxide nanoparticle surfaces. The immobilized enzyme showed superior resistance to UVC irradiation and temperature variations compared to its free form. Furthermore, storing the material simply by dispersing it in a buffer and keeping it in a refrigerator resulted in enzyme stability for up to 50 days. These features can be considered advantageous for developing wastewater treatments to degrade environmental contaminants.

When Lac-MNPs were combined with UVC/H_2_O_2_, this innovative approach achieved 91.9 ± 2.1% CBZ removal after 15 min of batch reaction under the conditions: [CBZ]_0_= 5 mg L^–1^, 20:1 H_2_O_2_:CBZ molar ratio, 42.5 W m^–2^ UVC irradiance, Lac-MNPs= 0.8 g L^–1^, and [ABTS] = 30 μmol L^–1^. In continuous operation mode, the developed laccase-photo-Fenton treatment under CCRD-optimized conditions showed 91.1% removal within a space-time of 10 min using 20:1 H_2_O_2_:CBZ molar ratio and 30 μmol L^–1^ ABTS as an inducer for laccase activity (R^2^ = 0.8707, p-value = 0.028).

The main degradation mechanisms of CBZ involve oxidation, epoxidation, hydroxylation, ring cleavage, hydrogen abstraction and decarboxylation. The remarkable stability, combined with the operational performance of the hybrid continuous flow laccase-photo-Fenton treatment, demonstrated its effectiveness and efficiency for rapid CBZ removal from water. Nevertheless, some transformation products were found to be potentially more harmful than the parent compound, indicating the need for *in vivo* testing the treated water to ensure its environmental compliance.

## Supplementary Material



## Data Availability

Data will be made available on request to the corresponding author.

## Notes

Additional experimental details, equations, methods and results, including (i) Schemes of the 3D-printed flat-plate reactor; (ii) Spectral molar absorption coefficients of carbamazepine; (iii) Performance of laccase under UVC irradiation; (iv) ABTS oxidation in continuous flow mode; (v) Continuous mode experiments for carbamazepine removal; (vi) Operation details of the liquid chromatography-tandem mass spectrometry for quantification of carbamazepine; (vii) Estimated ecotoxicity parameters by ECOSAR software (PDF)
