# Supplementary material for: Efficient Degradation of Carbamazepine in Continuous and Batch Modes by Laccase-Photo-Fenton-Intensified Hybrid Treatment
Source: ACS ES T Water. 2025 Nov 24;5(12):7253–66. doi: 10.1021/acsestwater.5c00676 (PMC12706775; doi:10.1021/acsestwater.5c00676)
Supplement: Supplementary file 1 [file ew5c00676_si_001.pdf]

SUPPLEMENTARY MATERIALS

Efficient degradation of carbamazepine in continuous and batch modes by laccase-photo-Fenton-intensified hybrid treatment

Natalia Klanovicz[a], Pratishtha Khurana[b], Bruno Ramos[c], Helen Treichel[d], Satinder Kaur Brar[b], Antonio Carlos Silva Costa Teixeira[a]*

[a] Research Group in Advanced Oxidation Processes (AdOx), Department of Chemical Engineering, Escola Politécnica, Universidade de São Paulo, São Paulo, 05508-080, Brazil;

[b] Department of Civil Engineering, Lassonde School of Engineering, York University, Toronto, ON M3N 3A7, Canada;

[c] Department of Chemical Engineering, Centro Universitário FEI, São Bernardo do Campo, 09850-901, Brazil;

[d] Laboratory of Microbiology and Bioprocesses, Universidade Federal da Fronteira Sul, Erechim, 99700-000, Brazil.

*Corresponding author email: acscteix@usp.br (Antonio C S C Teixeira).

A. SUPPLEMENTARY EQUATIONS

$$CBZ\ removal\ (\%) = \frac{[CBZ]_0 - [CBZ]_t}{[CBZ]_0} * 100 \quad \textbf{(S1)}$$

$$C_{15}H_{12}N_2O + 40\ H_2O_2 = 15\ CO_2 + 45\ H_2O + 2\ HNO_3 \quad \textbf{(S2)}$$

Where:

$[CBZ]_0$ = Initial concentration of carbamazepine (mg L^{-1})

$[CBZ]_t$ = Concentration of carbamazepine at time t (mg L^{-1})

B. SUPPLEMENTARY FIGURES

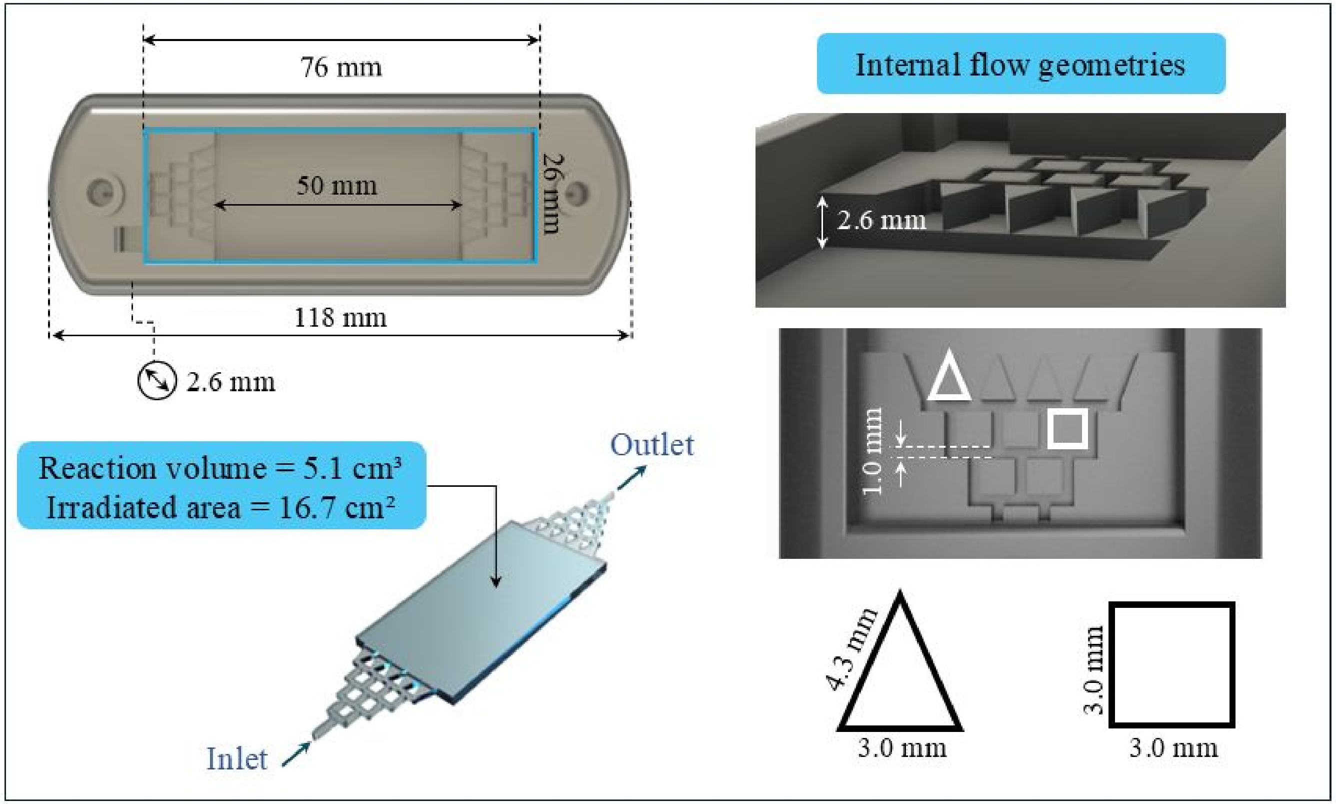

Fig. S1 – Details of the microstructured 3D-printed flat-plate reactor.

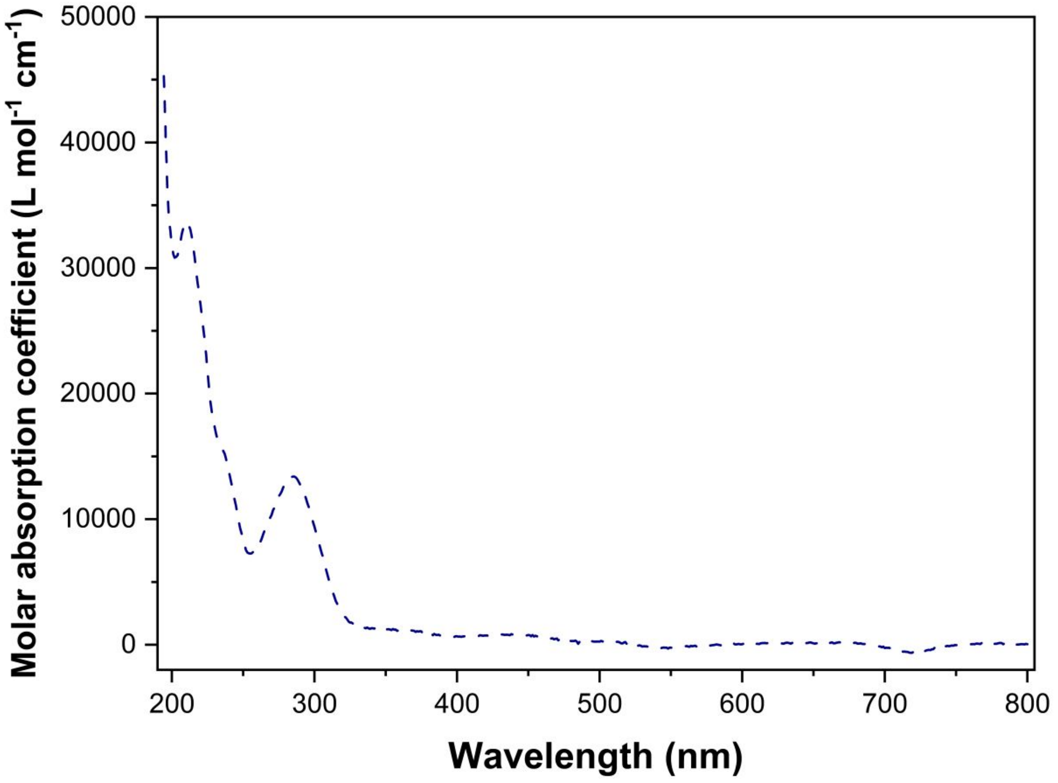

Fig. S2 – Spectral molar absorption coefficients of carbamazepine obtained using the Lambert-Beer Law.

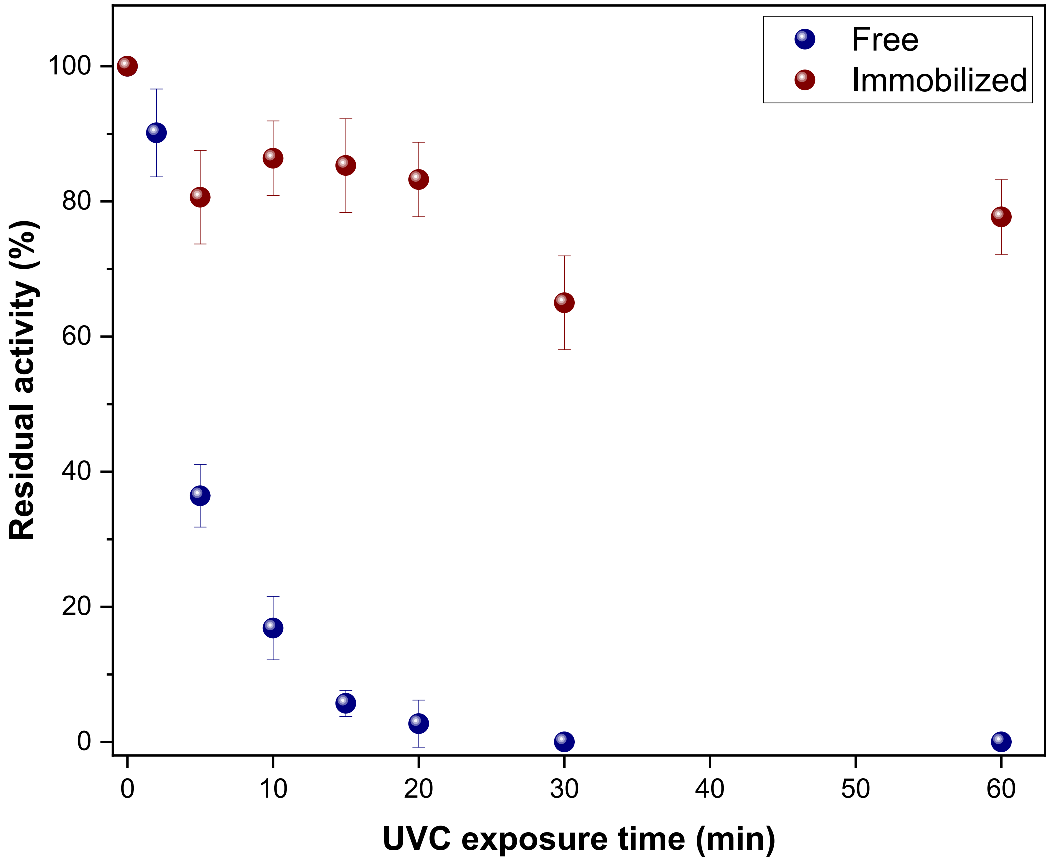

Fig. S3 – Performance of free and immobilized laccase under UVC irradiation at 42.5 W m^{-2}.

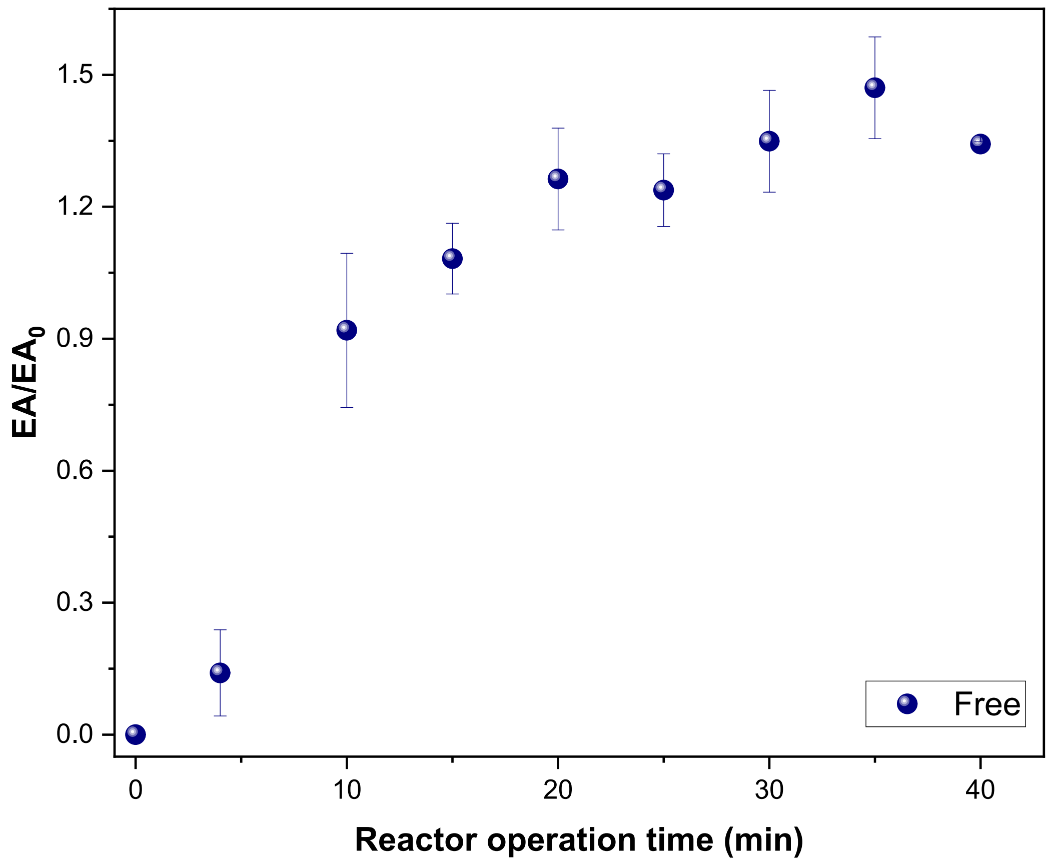

Fig. S4 – Results of ABTS oxidation in continuous flow mode for free laccase. Note: EA_0 corresponds to batch activity (set as 1.0).

C. SUPPLEMENTARY TABLES

Table S1 – Operation details of the liquid chromatography-tandem mass spectrometry (LCMS/MS) for quantifying carbamazepine (CBZ).

**Parameter**	**Specification**
Column	Nexcol C18 50mm × 2.1 mm × 1.8 µm
Eluants	A - Water with 0.1% formic acid<br>B - Acetonitrile with 0.1% formic acid
Gradient elution program	0 min, 10 % B<br>1 min, 10 % B<br>5 min, 70 % B<br>6.5 min, 70 % B<br>6.6 min, 10 % B<br>11 min, 10 % B
Flow	0.2 mL min^{-1}
Injection volume	10 µL
CBZ mass	Q1 - 237 m/z<br>Q3 - 194 m/z

Table S2 – Characteristics of TPs and estimated lethal, expected, and chronic doses for fish (F), daphnid (D), and green algae (GA) from ECOSAR calculations.

Compound	m/z	Chemical formula	SMILES code	Chemical and physical properties*			Acute toxicity*		Chronic toxicity*
				Molecular weight (g mol^{-1})	Water solubility (mg L^{-1})	Log K_{ow}	LC_{50}	EC_{50}	ChV
CBZ	237	C15H12N2O	NC(=O)N2c1ccccc1C=Cc3ccccc23	236.28	28.36	2.25	F_{96h} = NES<br>D_{48h} = 67.47	GA_{96h} = 55.26	F = 11.66<br>D = 7.01<br>GA = 15.23
TP1	267	C15H10N2O3	O=C(N)[N+]2c3ccccc3(C(=O)C(=O)c1ccccc12)	266.26	119.89	1.28	F_{96h} = NES<br>D_{48h} = NES	GA_{96h} = NES	F = 87.41<br>D = 41.66<br>GA = 65.72
TP2	271	C15H14N2O3	O=C(N)[N+]3c1ccccc1C(O)C(O)c2ccccc23	270.29	103.93	-0.21	F_{96h} = NES<br>D_{48h} = NES	GA_{96h} = NES	F = NES<br>D = NES<br>GA = NES
TP3	253	C15H12N2O2	O=C(N)[N+]3c1ccccc1C4OC4(c2ccccc23)	252.27	276.84	0.95	F_{96h} = 94.82<br>D_{48h} = NES	GA_{96h} = NES	F = 0.04<br>D = 32.24<br>GA = 217.59
TP4	253	C15H12N2O2	[H]C(=O)C3c1ccccc1[N+](C(=O)N)c2ccccc23	252.27	303.65	0.90	F_{96h} = 51.49<br>D_{48h} = 52.14	GA_{96h} = 27.14	F = 12.61<br>D = 0.47<br>GA = 8.98
TP5	210	C14H11NO	OC2=Cc1ccccc1[N+]c3ccccc23	209.25	5.94	3.17	F_{96h} = NES<br>D_{48h} = NES	GA_{96h} = NES	F = 1.69<br>D = 1.27<br>GA = 3.74
TP6	210	C14H11NO	O=CC3c1ccccc1[N+]c2ccccc23	209.25	14.74	2.71	F_{96h} = 5.83<br>D_{48h} = 4.97	GA_{96h} = 3.16	F = 0.58<br>D = 0.06<br>GA = 1.40
TP7	287	C15H14N2O4	O=C(N)[N+]3c1ccccc1C(O)C(O)c2cc(O)ccc23	286.29	431.59	-1.04	F_{96h} = NES<br>D_{48h} = NES	GA_{96h} = NES	F = NES<br>D = NES<br>GA = NES

Table S2 (Cont.) – Characteristics of TPs and estimated lethal, expected, and chronic doses for fish (F), daphnid (D), and green algae (GA) from ECOSAR calculations.

Compound	m/z	Chemical formula	SMILES code	Chemical and physical properties*			Acute toxicity*		Chronic toxicity*
				Molecular weight (g mol^{-1})	Water solubility (mg L^{-1})	Log K_{ow}			
TP8	236	C15H9NO2	O=C=[N+]2c3ccccc3(C=C(O)c1ccccc12)	236.25	0.52	4.24	F_{96h} = NES<br>D_{48h} = NES	GA_{96h} = NES	F = 0.24<br>D = 0.23<br>GA = NES
TP9	180	C13H9N	n(c(c(ccc1)cc2cccc3)c1)c23	179.22	22.70	3.32	F_{96h} = 9.63<br>D_{48h} = 6.18	GA_{96h} = 7.61	F = 1.09<br>D = 0.85<br>GA = 2.61
TP10	196	C13H9NO	c12nc3ccc(O)cc3cc1cccc2	195.22	51.44	2.84	F_{96h} = 28.32<br>D_{48h} = 17.38	GA_{96h} = 17.83	F = 3.03<br>D = 2.10<br>GA = 5.55
TP11	212	C13H9NO2	Oc1cc2cc3ccccc3([n+]c2(cc1(O)))	211.22	109.43	2.36	F_{96h} = 82.73<br>D_{48h} = 48.55	GA_{96h} = 41.47	F = 8.41<br>D = 5.19<br>GA = 11.69
TP12	228	C13H9NO3	Oc1cc2[n+]c3ccccc3(cc2(c(O)c1(O)))	227.22	101.00	2.30	F_{96h} = 100.44<br>D_{48h} = 58.63	GA_{96h} = 48.97	F = 10.14<br>D = 6.18<br>GA = 13.64
TP13	244	C13H9NO4	Oc1c(O)c(O)c2[n+]c3ccccc3(cc2(c1(O)))	243.22	212.57	1.82	F_{96h} = NES<br>D_{48h} = 162.06	GA_{96h} = 112.68	F = 27.81<br>D = 15.09<br>GA = 28.43
TP14	224	C14H9NO2	c(ccc1nc2cc3)cc1c(c2cc3)C(=O)O	223.23	4.75	3.20	F_{96h} = NES<br>D_{48h} = NES	GA_{96h} = NES	F = NES<br>D = NES<br>GA = NES
TP15	224	C14H9NO2	O=C2C(=O)c1ccccc1[N+]c3ccccc23	223.23	5.88	3.09	F_{96h} = NES<br>D_{48h} = NES	GA_{96h} = NES	F = 2.11<br>D = 1.56<br>GA = 4.46

* Estimated by ECOSAR software; Log K_{ow} = Octanol-water partition coefficient; LC_{50} = lethal concentration of a substance that can cause death in 50% of a test group; EC_{50} = expected concentration of a substance that can cause a specific adverse effect in 50% of a test group; ChV = chronic values; NES = no effects at saturation, reported when the effect level exceeds water solubility.
